# Drug‐resistant seizures associated with hyperinflammatory monocytes in FIRES

**DOI:** 10.1002/acn3.51755

**Published:** 2023-03-16

**Authors:** Charles L. Howe, Renee K. Johnson, Brittany L. Overlee, Jessica A. Sagen, Niyati Mehta, Raquel Farias‐Moeller

**Affiliations:** ^1^ Translational Neuroimmunology Lab Mayo Clinic Rochester Minnesota USA; ^2^ Department of Neurology Mayo Clinic Rochester Minnesota USA; ^3^ Center for MS and Autoimmune Neurology Mayo Clinic Rochester Minnesota USA; ^4^ Division of Experimental Neurology Mayo Clinic Rochester Minnesota USA; ^5^ Department of Neurology Medical College of Wisconsin Milwaukee Wisconsin USA; ^6^ Division of Child Neurology Medical College of Wisconsin Milwaukee Wisconsin USA

## Abstract

**Objective:**

Therapeutic strategies for patients with febrile infection‐related epilepsy syndrome (FIRES) are limited, ad hoc, and frequently ineffective. Based on evidence that inflammation drives pathogenesis in FIRES, we used ex vivo stimulation of peripheral blood mononuclear cells (PBMCs) to characterize the monocytic response profile before and after therapy in a child successfully treated with dexamethasone delivered intrathecally six times between hospital Day 23 and 40 at 0.25 mg/kg/dose.

**Methods:**

PBMCs were isolated from serial blood draws acquired during refractory status epilepticus (RSE) and following resolution associated with intrathecal dexamethasone therapy in a previously healthy 9‐year‐old male that presented with seizures following Streptococcal pharyngitis. Cells were stimulated with bacterial or viral ligands and cytokine release was measured and compared to responses in age‐matched healthy control PBMCs. Levels of inflammatory factors in the blood and CSF were also measured and compared to pediatric healthy control ranges.

**Results:**

During RSE, serum levels of IL6, CXCL8, HMGB1, S100A8/A9, and CRP were significantly elevated. IL6 was elevated in CSF. Ex vivo stimulation of PBMCs collected during RSE revealed hyperinflammatory release of IL6 and CXCL8 in response to bacterial stimulation. Following intrathecal dexamethasone, RSE resolved, inflammatory levels normalized in serum and CSF, and the PBMC hyperinflammatory response renormalized.

**Significance:**

FIRES may be associated with a hyperinflammatory monocytic response to normally banal bacterial pathogens. This hyperinflammatory response may induce a profound neutrophil burden and the consequent release of factors that further exacerbate inflammation and drive neuroinflammation. Intrathecal dexamethasone may resolve RSE by resetting this inflammatory feedback loop.

## Introduction

Prolonged seizures that are not controlled by treatment with benzodiazepines and intravenous antiseizure medications are characterized as refractory status epilepticus (RSE).^1^ Patients with such seizures require treatment with anesthetics to suppress the unremitting ictal activity.^2^ While many patients that present with RSE have a readily identified etiology,^3^ a sizable population develop prolonged RSE in the absence of any clear cause. New‐onset refractory status epilepticus (NORSE) is defined as the clinical presentation of RSE in a previously healthy individual with no history of seizures and no acutely identifiable structural, toxic, or metabolic cause.^4^ A subset of NORSE patients exhibit a prodromal febrile infection, with fever starting between 2 weeks and 24 h before seizure onset. This presentation is referred to as FIRES, for febrile infection‐related epilepsy syndrome.^4^, ^5^ While both adults and children can present with FIRES,^6^ there is a predilection for FIRES (as compared to NORSE, per se) in school‐aged children.^7^ Etiology is eventually identified in many NORSE patients after extended workup, with sporadic or paraneoplastic autoimmune encephalitis as the dominant cause.^8^ But a substantial number of cases remain cryptogenic.^9^

Outcomes in patients with FIRES and NORSE are generally poor, with greater than 10% mortality^10^ and long‐term neurological sequelae in survivors ranging from cognitive delay and impairment to severe and irreversible encephalopathic brain damage.^5^ Permanent drug‐resistant epilepsy is a nearly uniform outcome.^8^ We^11^, ^12^, ^13^, ^14^ and others^9^, ^10^, ^15^, ^16^, ^17^, ^18^, ^19^, ^20^, ^21^ have explored an inflammatory basis for RSE in NORSE patients. Given the febrile infection‐related component of FIRES we have also postulated a maladaptive inflammatory or autoinflammatory component to pathogenesis in these patients.^14^ Based on the limited correlation between serum and CSF inflammatory biomarker measurements and therapeutic response to immunomodulatory drugs such as anakinra and tocilizumab,^11^, ^13^, ^21^, ^22^, ^23^, ^24^, ^25^, ^26^, ^27^ we postulated that cellular response profiles might provide valuable insights into the underlying pathogenic mechanisms at play in patients with FIRES. To that end, we collected peripheral blood mononuclear cells (PBMCs) from a young male that presented with FIRES following Streptococcal pharyngitis. During the complicated therapeutic odyssey in this child we collected repeated PBMCs and biofluids in an effort to characterize changes in inflammatory response associated with treatment. Remarkably, this patient responded to intrathecal dexamethasone^28^ with a profound recovery and resolution of both inflammation and RSE.

## Materials and Methods

### Patient and healthy control biospecimens

All experiments were approved by the Mayo Clinic Institutional Review Board (#08‐007846, #19‐001219). Written informed consent was received from the subject's legally authorized representative. All methods were performed in accordance with the relevant guidelines and regulations. Whole blood was collected into EDTA tubes and serum separator tubes. Serum was processed locally, aliquoted, frozen, and shipped overnight on dry ice to the testing site. Whole blood was shipped overnight at ambient temperature to the testing site.^29^ Upon arrival the whole blood was assessed on a DxH 500 Hematology Analyzer (Beckman Coulter, B40601) to obtain complete blood counts and the leukocyte differential prior to experiments and peripheral blood mononuclear cells (PBMCs) were prepared as described below.

Control whole blood samples for immunophenotyping were collected from two healthy donors (males, 8‐ and 9‐years of age) processed under the same conditions used for the patient (i.e., blood held overnight in EDTA tubes at ambient temperature). Control whole blood for complete blood count and differential was collected from a healthy 5‐year‐old male. Control PBMCs for ex vivo stimulation were collected from a 7‐year‐old male under identical conditions. Healthy pediatric control serum samples were collected from 48 children (age range: 4.7–11.1 years; 27 females, 21 males). Control CSF samples were collected from nine adults with normal pressure hydrocephalus. Finally, blood was collected from the patient's biological parents and shipped to the testing site under the same conditions used for the patient.

### Whole blood immunophenotyping

Whole blood was incubated with 25% normal mouse serum (Jackson ImmunoResearch, 015–000‐120) to block non‐specific sites. After 5–10 min, primary antibody was added at 1:25 dilution: FITC anti‐CD16 (Biolegend, 302006), PE anti‐CD14 (Tonbo, 50‐0149‐T100), PerCPCy5.5 anti‐CD3 (Biolegend, 300328), APC anti‐HLADR (Biolegend, 307610), and AlexaFluor700 anti‐CD66b (Biolegend, 305114). Samples were incubated at room temperature for 20 min protected from light and then incubated with 1 mL of VersaLyse (Beckman Coulter, A09777) at room temperature for 20 min to remove red blood cells. Tubes were centrifuged at 400*g* for 3 min at room temperature, supernatant was aspirated, and cells were resuspended in 1 mL of room temperature PBS containing 1% BSA (SeraCare, 1900‐0016). Tubes were centrifuged again at 400*g* for 3 min, supernatant was aspirated, and cells were resuspended in 300 μL of ice‐cold freshly prepared 0.2% paraformaldehyde (Sigma, 158127) prepared in PBS.

### Isolation of PBMCs

PBMCs were isolated using Leucosep tubes (Greiner Bio‐One, 163290P) and Lymphoprep (STEMCELL Technologies, 7801), per manufacturer directions. Following centrifugation at 800*g* for 15 min at 20°C in a swinging bucket rotor with no brake, the PBMC‐enriched layer was diluted to 50 mL with RPMI (Invitrogen, 11875‐093), and then centrifuged at 250*g* for 10 min at 20°C in a swinging bucket rotor. The cell pellet was resuspended in RPMI containing 1% human serum (Sigma, H6914).

### Cryopreservation and cryorecovery

Freshly prepared PBMCs were resuspended at 5 × 10^6^ cells per vial in RPMI containing 10% human serum and 10% dimethyl sulfoxide (DMSO), frozen at −1°C/min, and stored in vapor phase above liquid nitrogen. Cells were cryorecovered by rapid thaw at 37°C followed by dilution to 50 mL in RPMI containing 10% human serum. Cells were rested for 30 min at 37°C without agitation, then pelleted by centrifugation at 250 *g* for 5 min at 20°C in a swinging bucket rotor.

### Ex vivo stimulation

Following cryorecovery, cells were resuspended in RPMI containing 1% human serum, counted, and plated at 2 × 10^5^ cells per well in 200 μL in ultra‐low attachment U‐bottom 96‐well plates (Corning, 7007). Cells were stimulated overnight with a pan‐bacterial cocktail comprised of lipopolysaccharide from Escherichia coli O26:B6 (LPS) (Sigma, L2654) at 1 μg/mL and heat‐killed *Staphylococcus aureus* (HKSA) (InvivoGen, tlrl‐hksa) at 10^8^ cells/mL, or with a pan‐viral cocktail comprised of high molecular weight (HMW) poly(I:C) (InvivoGen, tlrl‐pic), Lyovec encapsulated HMW poly(I:C) (InvivoGen, tlrl‐piclv), single‐stranded Poly(U) naked RNA (InvivoGen, tlrl‐sspu), and Lyovec encapsulated ssPoly(U) (InvivoGen, tlrl‐lpu), each at 1 μg/mL final concentration. Concentrated cocktails were added to stimulated cells at 1:100 dilution. Unstimulated cells received an equivalent amount of water as vehicle. Cells were incubated for 24 h at 37°C with gentle agitation at 80 rpm on an orbital shaker (Eppendorf, M1190‐0000) housed inside a standard tissue culture incubator (5% CO_2_, humidified). Following stimulation, cells were centrifuged at 400*g* for 5 min at room temperature and supernatants were collected, clarified at >16,000*g* for 5 min, and stored at −20°C for analysis.

### Multiplexed cytometric bead array and enzyme‐linked immunosorbent assay

Serum and CSF were assessed using the Human Inflammatory Cytokine kit (BD, 551811) (CXCL8, IL1β, IL6, IL10, IL12p70, TNFα) and the Human Chemokine kit (BD, 552990) (CCL2, CCL5, CXCL8, CXCL9, CXCL10). Supernatants (50 μL) from stimulated PBMCs were analyzed using the Human Soluble Protein Master Buffer Kit (BD, 558264) and cytometric beads reactive for IL1α (BD, 560153), IL1β (BD, 558279), IL‐6 (BD, 558276), TNFα (BD, 558273), CCL2 (BD, 558287), and CXCL8 (BD, 558277). Standard curves were generated in FCAP Array (BD) and manually post‐processed in Prism (GraphPad) and Excel (Microsoft). Samples were also analyzed using commercial ELISAs against HMGB1 (Tecan, 30164033) and S100A8/A9 (R&D, DY8226‐05), following manufacturer's directions.

### Data analysis

Flow cytometric data were analyzed in FlowJo v10.8.1 (BD). Descriptive statistics and standard curves were calculated in Prism 9.4.0. Graphs were generated in Prism and then modified in Adobe Illustrator for layout and consistency.

## Results

### Clinical summary

**Figure 1:**
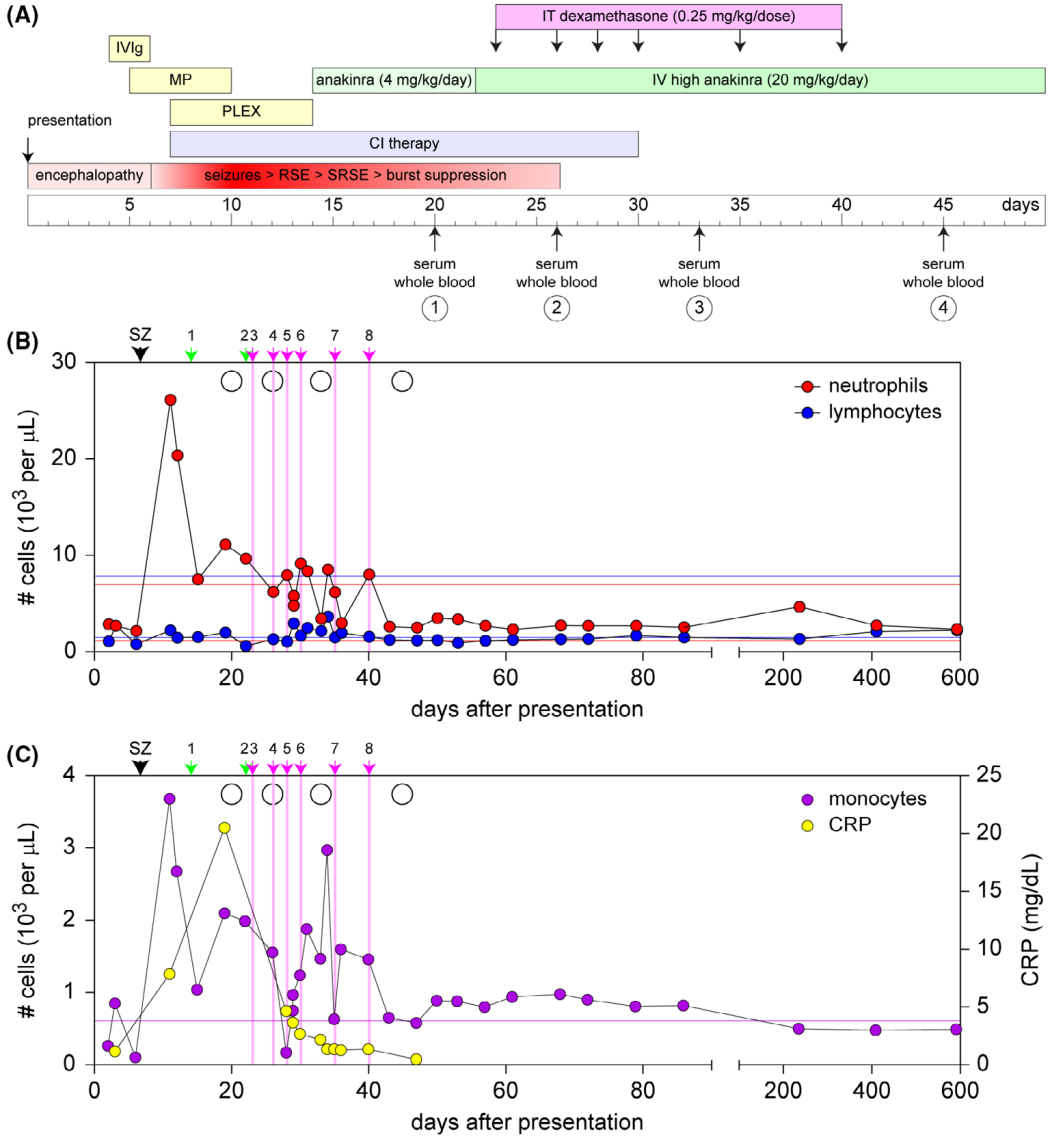
Schematic timeline of sample collection timepoints relative to treatments and clinical analysis of complete blood counts. (A) Intravenous immunoglobulin (IVIg), methylprednisolone (MP), and plasma exchange (PLEX) therapies were initiated within days of presentation to no effect. Continuous infusion (CI) therapy with midazolam and ketamine was initiated at Day 7 with escalating doses and titration to achieve suppression of RSE and SRSE; pentobarbital was added at Day 10. Anakinra was initiated at day 14 and the dose was escalated on Day 22. Intrathecal dexamethasone was started on Day 23, with further injections on days 26, 28, 30, 35, and 40. Continuous infusion therapy weaning was successfully completed by Day 30 without return of RSE. Serum and whole blood samples were collected on days 20 (pre‐IT dexamethasone), 26, 33, and 45. (B) Analysis of complete blood counts revealed a large increase in neutrophil burden coincident with the onset of RSE. During the same timeframe lymphocyte levels were low. While neutrophil numbers decreased, they remained above normal range until completion of the i.t. dexamethasone therapy. SZ = seizure onset. 1 = start of low‐dose anakinra; 2 = start of high‐dose anakinra; 3–8 = intrathecal dexamethasone treatments. Neutrophils are shown in red; lymphocytes are shown in blue. The thin red lines represent upper and lower boundaries of normal neutrophil counts; thin blue lines represent boundaries of normal lymphocyte counts. Empty circles represent research blood draws. (C) Monocyte numbers also increased coincident with RSE onset. In addition, levels of C‐reactive protein (CRP) increased to an exceptionally high level during SRSE and prior to i.t. dexamethasone therapy. Monocytes are shown in purple; CRP is shown in yellow. Left axis refers to monocyte counts; right axis refers to CRP levels. Thin purple line represents the upper bound of normal monocyte numbers. Other symbols as in panel B.

A previously healthy 9‐year‐old male presented with acute encephalopathy marked by crying and confusion in the context of Streptococcal pharyngitis with fever that had resolved 2 days before presentation. Temperature at presentation was 36.8°C. Initial workup revealed mild pleocytosis with 30 white blood cells per microliter in the CSF (76% lymphocytes, 11% neutrophils, 13% monocytes). CSF protein levels were normal (29 mg/dL). MRI of the brain was normal. Broad spectrum antibiotics and acyclovir were implemented. The patient's initial EEG was marked by bilateral slowing that was more pronounced in the left temporal region. Six days after presentation the patient began having seizures that progressed to convulsive status epilepticus, with focal to bilateral seizures that were refractory to bolus treatment with conventional anti‐seizure medications (Fig. 1A). Further treatments with enteral ketogenic diet, intravenous immunoglobulin, intravenous methylprednisolone, and plasmapheresis were unsuccessful. The EEG was characterized by left posterior initiation with spread to the left temporal and parietal regions and to the contralateral hemisphere. While most seizures originated from the left hemisphere, about 25% started in the right posterior quadrant. During the hyperacute phase the seizures were focal to bilateral tonic clonic lasting approximately 1.5–3 min, becoming progressively longer, up to 5 min. The initial frequency was about 8–10 per day, progressively increasing to >20% of the EEG epoch until burst suppression was achieved with continuous anesthetic infusion (midazolam and ketamine started on day 7; pentobarbital added on day 10). Every attempt to wean the burst suppression during the first 23 days resulted in return of seizures. Comprehensive investigation identified no structural, infectious, genetic, metabolic, or toxic etiologies. The child was therefore diagnosed with FIRES. Subsequent autoantibody screening of serum and CSF was negative.

Due to ongoing super‐refractory status epilepticus (SRSE) the patient's therapeutic regimen was escalated to include anakinra at 4 mg/kg/day starting on hospital Day 14. This was increased to 20 mg/kg/day provided intravenously every 6 h starting on Day 22. SRSE persisted and on Day 23 the child was treated with intrathecal (i.t.) dexamethasone via lumbar puncture at a pulse dose of 0.25 mg/kg. Five further doses were delivered on hospital days 26, 28, 30, 35, and 40, with successful weaning from continuous anesthetic infusion by Day 30 and resolution of RSE (Fig. 1A). EEG at this point exhibited patterns in the ictal‐interictal continuum but no definitive seizures were noted. After several weeks of surveillance EEG a few short electrographic seizures lasting less than 1 min were noted. The patient was successfully discharged after 89 days of hospitalization with a favorable neurologic outcome despite global cerebral atrophy. Medically refractory epilepsy was subsequently treated by surgical resection of tissue in the left occipital lobe which exhibited mild reactive gliosis on pathology. The child remains seizure free 6 months after the procedure.

### Biospecimen summary and clinical blood counts

Serum and whole blood samples were collected at 20 (draw 1), 26 (draw 2), 33 (draw 3), and 45 (draw 4) days after presentation (Fig. 1A). Draw 1 represents the peripheral state of the patient prior to high dose anakinra and i.t. dexamethasone and during ongoing severe drug‐refractory seizures. Draw 2 represents the peripheral state of the patient at the start of continuous infusion therapy weaning and resolution of RSE. Draws 3 and 4 represent the post‐resolution state.

Clinical analysis of white blood cell numbers revealed a large increase in neutrophils and monocytes coincident with seizure onset in the patient (Fig. 1B,C). At the time the patient entered status epilepticus his neutrophil count was 26240 cells per μL (normal range 1100–6900 cells/μL) and his monocyte count was 3680 cell per μL (normal range 0–600 cells/μL). In parallel, his lymphocyte count was at the lower end of normal (2240 cells/μL; normal range 1400–7800 cells/μL). Notably, at the time of presentation the patient had 0.7 mg/dL C‐reactive protein (CRP) in blood, as assessed by clinical testing. This marker of inflammation increased to 7.4 mg/dL at the onset of RSE and continued to increase to 20.1 mg/dL just prior to the initiation of i.t. dexamethasone.

In addition to the clinical blood counts, the whole blood research samples were analyzed for complete blood counts with differential immediately upon receipt. In agreement with the clinical measurements, prior to seizure resolution (draw 1) the patient had a large neutrophil and monocyte burden coupled to low lymphocyte counts (Table 1). Indeed, the neutrophil‐to‐lymphocyte ratio^30^, ^31^, ^32^ at this timepoint was nearly 15 and the monocyte‐to‐lymphocyte ratio^33^, ^34^ was over two, indicative of profound systemic inflammation. Both ratios were reduced by draw 2 and remained within or near normal range over subsequent draws.

**Table 1:** Complete blood count and differential.

	Control^a^	Patient draw 1	Patient draw 2	Patient draw 3	Patient draw 4	Normal range^b^	
Lymphocytes	2650	880	2820	1250	1260	1000–4800	#/μL
Monocytes	420	1950	1000	630	460	300–900	#/μL
Neutrophils	2440	13,020	5980	3890	2750	1700–7000	#/μL
Eosinophils	70	110	90	230	160	100–500	#/μL
Basophils	10	100	30	40	40	0–300	#/μL
RBC	4.17	3.23	3.01	2.97	3.54	3.8–5.7	×106/μL
Hemoglobin	11.88	9.85	12.02	9.61	12.00	10.9–16.6	g/dL
Hematocrit	34.9	32.2	28.8	29.5	36.0	33–50	%
MCV	83.8	99.7	95.8	99.3	101.8	80–100	fL
MCH	28.5	30.5	39.9	32.4	33.9	27–31	pg
Platelets	358	670	687	413	473	135–450	×103/μL
NLR	0.9	14.8	2.1	3.1	2.2	0.8–1.9	
MLR	0.2	2.2	0.4	0.5	0.4	0.2–0.5	

Abbreviations: MCH, mean corpuscular hemoglobin; MCV, mean corpuscular volume; MLR, monocyte‐to‐lymphocyte ratio; NLR, neutrophil‐to‐lymphocyte ratio; RBC, red blood cells.

^a^ Control subject was healthy male, 5 years of age.

^b^ Normal ranges established in our lab.

### Serum inflammatory profile

**Figure 2:**
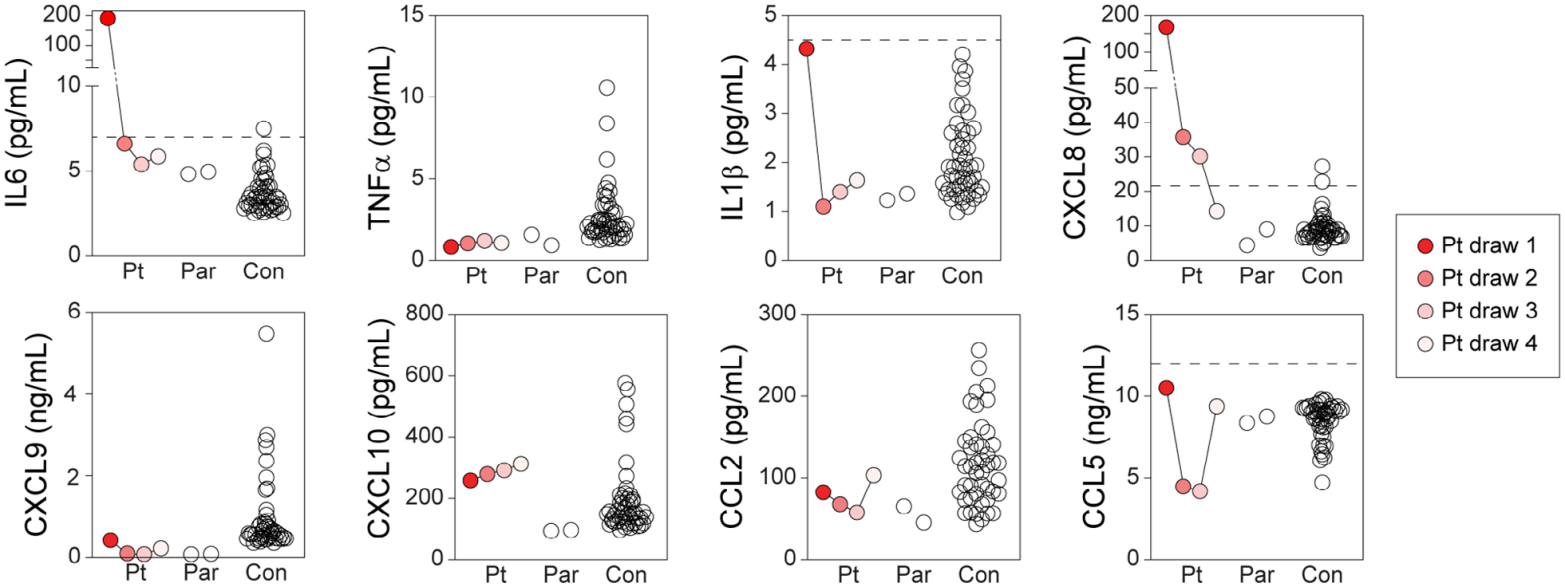
Serum inflammatory cytokine and chemokine levels. Levels of IL6, TNFα, IL1β, CXCL8, CXCL9, CXCL10, CCL2, and CCL5 were measured in serum collected at four timepoints using multiplexed cytometric bead arrays. Draw timepoints are color‐coded as shown in the legend. Dashed line represents 3 standard deviations above healthy control mean; if not shown, 3 SD level is off the graph. Each symbol represents a serum sample. Pt = patient; Par = patient's parents; Con = pediatric healthy controls.

Cytokine and chemokine measurements in the patient's serum were compared to samples collected from the parents and processed under similar conditions (Fig. 2). Measurements were also compared to healthy pediatric subjects and analytes were considered elevated if greater than 3 standard deviations above the control mean. Notably, IL6 (186.7 pg/mL) was 50‐fold higher than healthy control (3.7 ± 1.1 (SD) pg/mL; *n* = 48) and CXCL8 (170.6 pg/mL) was 18‐fold higher than control (9.5 ± 4.2 (SD) pg/mL; *n* = 48) in serum from draw 1 (Fig. 2). IL6 levels dropped to normal range and CXCL8 levels were substantially reduced (although still greater than 3 SD above control) by draw 2. The IL1β level in draw 1 (4.3 pg/mL) was at the upper range of healthy control (2.1 ± 0.8 (SD) pg/mL; *n* = 48) but was not elevated more than 3 SDs. The alarmins HMGB1 (27.2 ng/mL) and S100A8/A9 (9385 ng/mL) were also elevated more than 3 SD above control levels (HMGB1: 5.6 ± 3.3 (SD); *n* = 106; S100A8/A9: 1170 ± 528 (SD); *n* = 106) in draw 1 serum and were normalized after treatment. IL6 levels were also elevated more than 3 SD in the CSF prior to i.t. dexamethasone (80.8 pg/mL vs. 7.6 ± 5.6 (SD); *n* = 9) and normalized following treatment (4.9 pg/mL).

### Monocytic response profile prior to high dose anakinra and intrathecal dexamethasone

**Figure 3:**
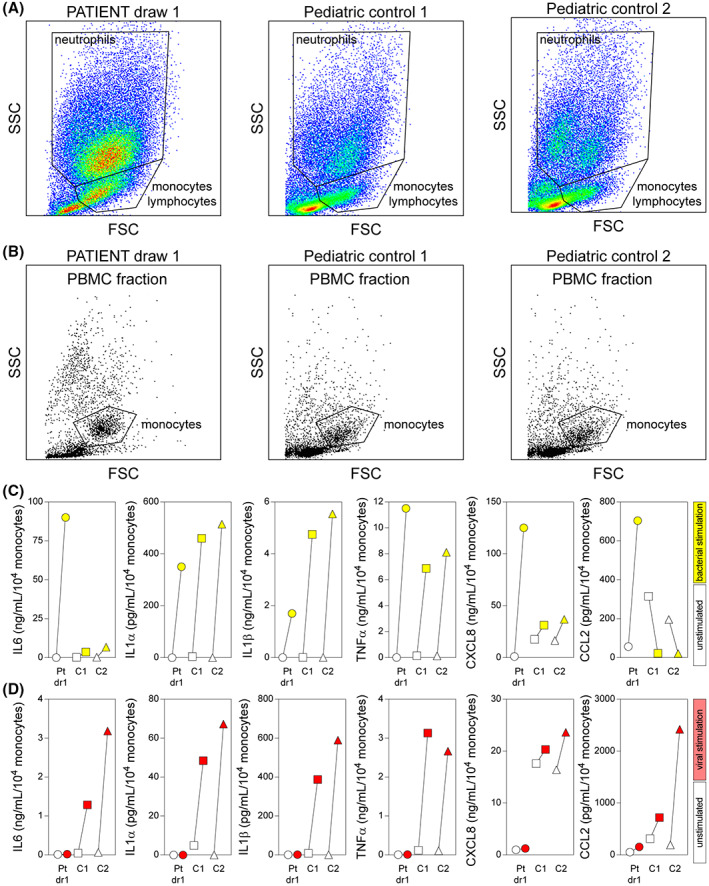
Ex vivo stimulation of freshly prepared PBMCs collected prior to i.t. dexamethasone. (A) Flow cytometric analysis of whole blood from the patient at draw 1 and from two age‐matched healthy controls (pediatric control 1 = 8‐year‐old male; pediatric control 2 = 9‐year‐old male). Gates outline populations based on scatter profile. (B) Flow cytometric analysis of PBMCs isolated by gradient centrifugation from whole blood in the same subjects. The monocyte gate was used to count cells for normalization and is based on back‐gating from HLADR^+^CD66b^−^CD14^+^ monocytes. (C) 2 × 10^5^ PBMCs were stimulated ex vivo with bacterial (LPS + HKSA) ligands for 24 h. Inflammatory cytokines and chemokines were measured in clarified supernatants using multiplexed cytometric bead arrays and induced levels (yellow) are shown relative to spontaneous release (unfilled symbols) measured in unstimulated wells. (D) 2 × 10^5^ PBMCs were stimulated ex vivo with viral (poly(I:C), LyoVec‐poly(I:C), ss_poly(U), LyoVec‐ss_poly(U)) ligands for 24 h. Inflammatory cytokines and chemokines were measured and induced levels (red) are shown relative to spontaneous release (unfilled symbols) measured in unstimulated wells. Pt dr 1 = patient draw 1; C1 = pediatric control 1; C2 = pediatric control 2.

Blood was collected from the patient (draw 1, prior to i.t. dexamethasone) and from two healthy age‐matched controls (pediatric control 1 = 8‐year‐old male; pediatric control 2 = 9‐year‐old male) and analyzed by flow cytometry (Fig. 3). Consistent with the CBC/differential findings, the patient exhibited a pronounced neutrophil population (Fig. 3A). Gradient purification of PBMCs revealed a slight upward shift in side scatter in the monocyte population but otherwise normal‐looking cells (Fig. 3B). The monocytic stimulus–response profile was assessed by incubating 2 × 10^5^ PBMCs in RPMI containing 1% human serum for 24 h in the presence or absence of a cocktail of bacterial ligands (LPS + HKSA) or viral ligands (poly(I:C), LyoVec‐poly(I:C), ss_poly(U), LyoVec‐ss_poly(U)). The bacterial ligand cocktail was chosen to broadly capture the responses induced by gram‐negative bacteria (LPS binding to Toll‐like receptor 4 (TLR4)) and gram‐positive bacteria (HKSA binding to TLR2). The viral ligand cocktail was selected to model double‐stranded RNA viruses that bind TLR3 (poly(I:C)) and TLR7/8 (ss_poly(U)). In addition, a critical component of innate viral sensing occurs at receptors within the cytosol and in the endosomal pathway. Therefore, we included both naked viral agonists and ligands complexed with the cationic lipid LyoVec, a molecule that facilitates cellular uptake.^35^, ^36^, ^37^

Following stimulation, supernatants were clarified and cytokine and chemokine levels were measured by multiplexed cytometric bead array. In response to bacterial stimulation and relative to the pediatric healthy controls, the patient exhibited exaggerated release of IL6, increased production of CXCL8, equivalent release of IL1α and TNFα, and slightly decreased release of IL1β (Fig. 3C). In contrast to controls, which exhibited suppression of CCL2 release below spontaneous levels in response to bacterial ligand stimulation, the patient's PBMCs showed a large increase in CCL2 release. In response to viral stimulation, the patient exhibited essentially no release above spontaneous levels for any of these factors. These findings suggest that the patient was hyper‐responsive to bacterial ligands, especially with regard to production and release of IL6 and CXCL8 (the factors elevated in serum at this timepoint), while simultaneously hypo‐responsive to viral stimuli.

### Peripheral blood immunophenotype

**Figure 4:**
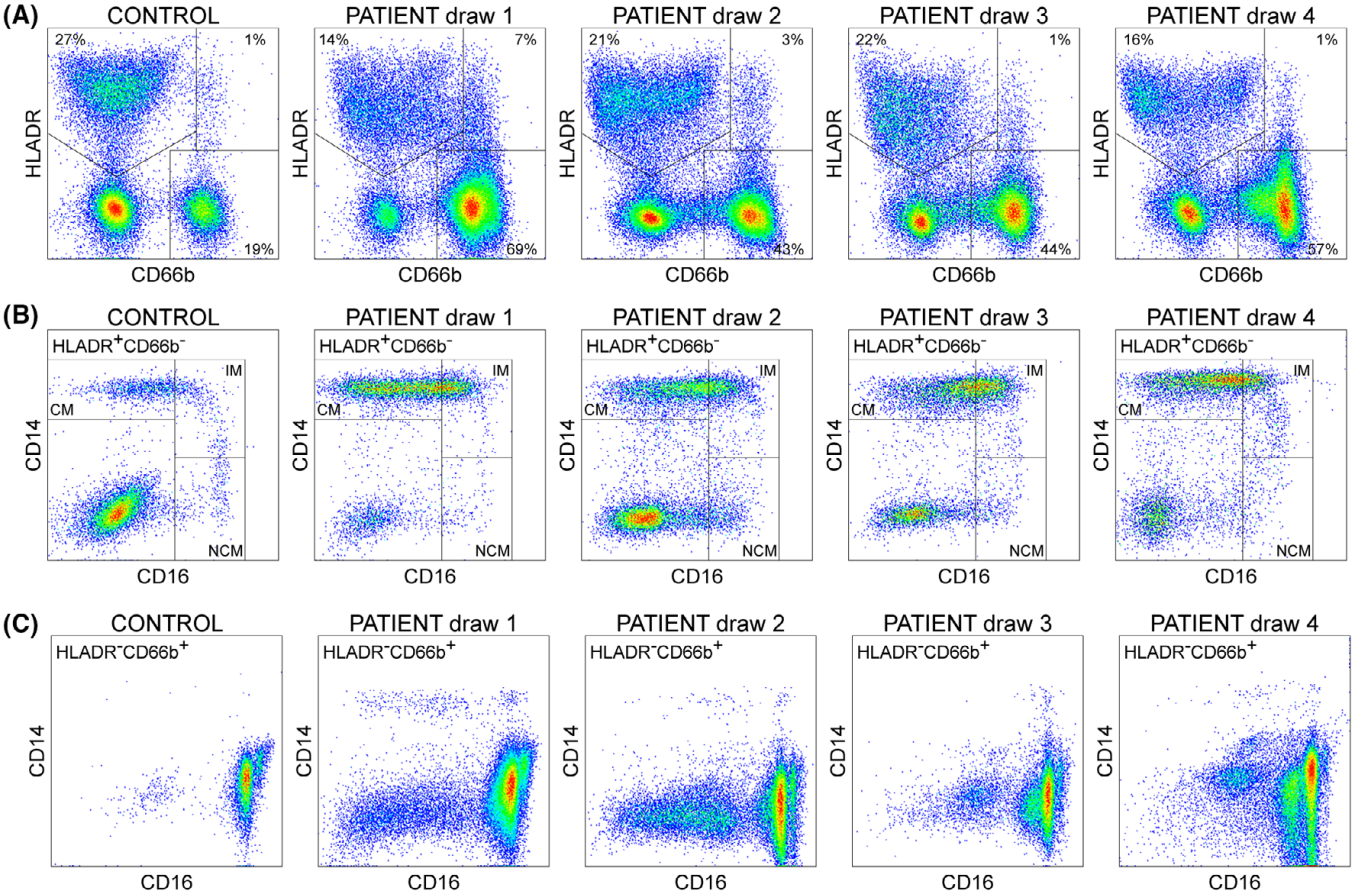
Whole blood immunophenotype. Whole blood was analyzed by flow cytometry after all four draws from the patient and compared to a pediatric healthy control (7‐year‐old male). (A) Singlets identified by forward scatter area and height were gated on HLADR and CD66b. Percentages of each population are shown in the respective gate. (B) The HLADR^+^CD66b^−^ population was further gated on CD14 and CD16 to reveal CD14^+^CD16^−^ classical monocytes (CM), CD14^+/mid^CD16^lo/mid^ inflammatory monocytes (IM), and CD14^lo/neg^CD16^mid^ non‐classical monocytes (NCM). (C) The HLADR^−^CD66b^+^ population was likewise gated on CD14 and CD16 to reveal CD14^lo/neg^CD16^hi^ neutrophils.

Blood collected from the patient at each draw timepoint was analyzed by flow cytometry to characterize the immunophenotype (Fig. 4). The patient pattern was compared to that observed in a healthy 7‐year‐old male. The overall pattern of HLADR and CD66b labeling indicated dispersion of the HLADR^+^CD66b^−^ population and over‐representation of the HLADR^−^CD66b^+^ population (Fig. 4A). The HLADR^+^CD66b^−^ population was further refined using CD14 and CD16 to identify CD14^+^CD16^−^ classical monocytes (CM), CD14^+/mid^CD16^lo/mid^ inflammatory monocytes (IM), and CD14^lo/neg^CD16^mid^ non‐classical monocytes (NCM) (Fig. 4B). In contrast to the healthy control (CM = 66%; IM = 20%; NCM = 14%), the ratio of classical monocytes to non‐classical was altered in the pre‐treatment draw (CM = 80%; IM = 19%; NCM = 1%) and did not completely normalize at any timepoint. Moreover, the number of CD16^mid^ inflammatory monocytes was increased in the pre‐treatment draw relative to control and remained elevated across draws. Likewise, the proportion of HLADR^−^CD66b^+^ granulocytes in the patient remained elevated across all four timepoints relative to the control (Fig. 4C). The high level of granulocytes in draw 1 is consistent with the CBC/differential at this timepoint (Table 1), but the over‐representation of this population at later timepoints is at odds with the blood count. In addition, the presence of CD14^−^CD16^lo^ cells in the HLADR^−^CD66b^+^ population was observed at every timepoint whereas only a very small number of such cells are ever observed in healthy controls. These findings suggest that despite resolution of seizures and systemic inflammation in the patient following i.t. dexamethasone, there was a persistent alteration in the phenotype of monocytes and neutrophils.

### Monocytic response profile after treatment

**Figure 5:**
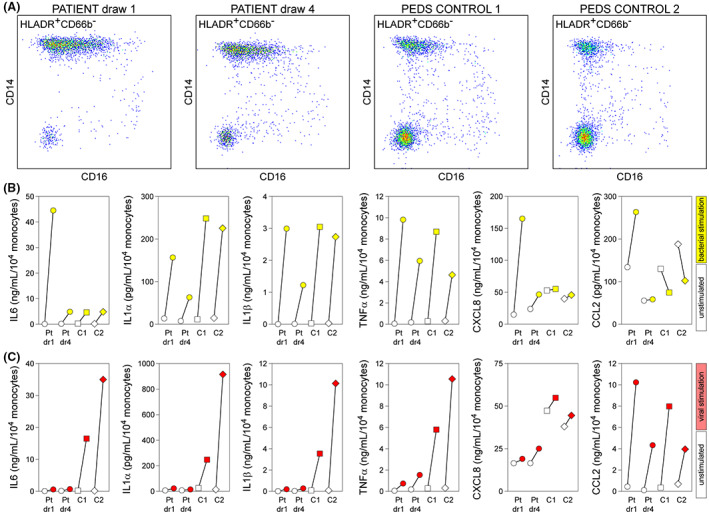
Ex vivo stimulation of cryorecovered PBMCs collected before and after therapy. (A) Immunophenotype of patient and control cryorecovered HLADR^+^CD66b^−^CD14^+^ monocytes used for ex vivo stimulation. (B) 2 × 10^5^ PBMCs were stimulated ex vivo with LPS + HKSA for 24 h. Inflammatory cytokines and chemokines were measured in clarified supernatants using multiplexed cytometric bead arrays and induced levels (yellow) are shown relative to spontaneous release (unfilled symbols) measured in unstimulated wells. (C) 2 × 10^5^ PBMCs were stimulated ex vivo with poly(I:C) + LyoVec‐poly(I:C) + ss_poly(U) + LyoVec‐ss_poly(U) for 24 h. Induced responses (red) are shown relative to spontaneous release (unfilled symbols). Pt dr 1 = patient draw 1; Pt dr 4 = patient draw 4; C1 = pediatric control 1; C2 = pediatric control 2.

Cryopreserved PBMCs isolated from whole blood collected prior to i.t. dexamethasone (Day 20) and at Day 45 were used for analysis of ex vivo stimulation responses. Responses were compared to cryopreserved cells from the same pediatric controls used in Fig. 3. Cryorecovered cells from draw 1 (Fig. 5A) maintained a phenotype similar to that observed in the fresh PBMCs (Fig. 4B). The monocytic response profiles triggered by bacterial ligands (LPS + HKSA) (Fig. 5B) and viral ligands (poly(I:C), LyoVec‐poly(I:C), ssPoly(U), LyoVec‐ssPoly(U)) (Fig. 5C) were measured as in Fig. 3. Cryorecovered cells from draw 1 maintained the same hyper‐responsive release of IL6 and CXCL8 in response to LPS + HKSA that was observed in fresh PBMCs (Figs. 3C and 5B). The aberrant increase in CCL2 release was also maintained. Notably, cryorecovered PBMCs from draw 4 were no longer hyper‐responsive, with stimulated IL6 and CXCL8 levels equivalent to healthy control responses. Indeed, the stimulated response to LPS + HKSA in draw 4 PBMCs was blunted for IL1α and IL1β relative to controls. In parallel, the hypo‐responsive profile induced in PBMCs from draw 1 in response to viral ligands was maintained in the cryorecovered cells (Fig. 5C). Remarkably, this response remained attenuated in cryorecovered PBMCs from draw 4. These findings indicate that the patient exhibited a transient hyper‐responsiveness to bacterial ligands that was not maintained following therapy but a hypo‐responsiveness to viral ligands that persisted. The normalization of the bacterial ligand response is consistent with the return of IL6 and CXCL8 levels to healthy control levels in serum at the draw 4 timepoint.

## Discussion

A child diagnosed with FIRES requiring continuous infusion of midazolam, ketamine, and pentobarbital to suppress drug‐refractory status epilepticus did not respond to IVIg, PLEX, systemic methylprednisolone, or anakinra. Analysis of serum and CSF revealed high levels of IL6, CXCL8, and HMGB1 despite these conventional therapies. In parallel, ex vivo stimulation of peripheral blood monocytes revealed exaggerated IL6 and CXCL8 release in response to bacterial exposure. However, this hyperinflammatory phenotype resolved following an increase in anakinra dose and initiation of intrathecal dexamethasone,^28^ and the patient was successfully weaned from the continuous sedative infusion therapy. The timing of the increase in anakinra and delivery of the first dose of intrathecal dexamethasone makes it impossible to assign therapeutic effect to only one of these interventions, but two factors argue for a dominant effect of the dexamethasone. First, IL6, CXCL8, and HMGB1 levels were elevated in serum and CSF while the patient was receiving low dose anakinra. The aberrant monocyte response was also measured during ongoing low dose anakinra therapy. Second, while serum IL1β levels decreased following therapy, the initial level was not elevated more than 3 standard deviations above the pediatric control mean and was within the healthy control range. Thus, while it is possible that the initial dose of anakinra was insufficient and increasing this drug alone would have resolved the inflammatory phenotype, we think that intrathecal dexamethasone was the key intervention that led to normalization of IL6, CXCL8, and HMGB1 and restoration of normal monocytic responses induced by exposure to bacteria. Moreover, even if anakinra or the combination of anakinra and i.t. dexamethasone was the key therapeutic approach that led to RSE resolution, the critical primary pathogenic outcome remains the resolution of hyper‐inflammatory monocytic responses.

The mechanism by which intrathecal dexamethasone mediated these effects is unclear. Initial therapy with high dose methylprednisolone did not alter seizures in the patient. In contrast to long‐acting dexamethasone, methylprednisolone is an intermediate‐acting steroid with a lower relative potency,^38^ but in general these corticosteroids show equivalent anti‐inflammatory efficacy when normalized for dose.^39^ Methylprednisolone concentrates in the lung following intravenous delivery, resulting in higher lung‐to‐plasma ratios relative to intravenous dexamethasone.^40^ Coupled with the different delivery compartments (i.v. vs. i.t.), this reduction in bioavailability may mean that either corticosteroid, if delivered at a dose and in a manner that enhanced the impact on circulating leukocytes, would have exerted therapeutic effect. Previous evidence indicates that intravenous delivery of corticosteroid results in rapid entry into CSF and the level achieved in CSF is essentially equivalent to the concentration of diffusible steroid present in plasma at any given time.^41^ Likewise, intracisternal delivery of corticosteroid resulted in rapid transfer into plasma, again essentially at a level equivalent to the diffusible concentration within the CSF. Notably, however, there may be enhanced sequestration of intrathecal corticosteroid in such a manner as to create a slow‐release phenomenon in which steroid is continuously detectable for many days in plasma after a single bolus delivery to CSF.^41^ This means that intrathecal delivery of corticosteroid may serve as a long‐lasting source that maintains a more persistent therapeutic effect than can be achieved with intravenous delivery. Such a model would argue that continuous slow intravenous delivery would phenocopy the intrathecal effect, perhaps with far better safety and adherence profiles.

Regardless of delivery site, corticosteroid therapy exerts effects on the peripheral immune system that may explain the reduction in systemic and CSF levels of inflammatory factors. Glucocorticoids bind to and dimerize the glucocorticoid receptor expressed in the majority of somatic cells, leading to nuclear translocation and transcriptional changes.^42^ Binding of the occupied receptor to glucocorticoid response elements in DNA results in trans‐activation of numerous anti‐inflammatory genes, including inhibitory kinase B, IL10, and annexin‐A1.^43^, ^44^ More powerfully, the occupied glucocorticoid receptor binds and inactivates transcription factors and transcriptional activators such as NFκB and AP‐1, resulting in trans‐repression of IL6, CXCL8, IL1β, and TNFα, among other inflammatory genes.^43^, ^44^ Thus, dexamethasone may have inhibited expression of the genes driving production of the inflammatory cytokines and chemokines elevated in the patient. Initial experiments testing the impact of dexamethasone on this patient's ex vivo response profile validated this concept but also revealed unexpected complexity in the steroid‐induced effect (data not shown). Future studies exploring the differential effect of dexamethasone and methylprednisolone using our ex vivo stimulation platform may provide insights into tailoring the corticosteroid therapy to maximize efficacy.

In addition to inhibiting inflammatory cytokine and chemokine production, dexamethasone may have reduced monocyte and neutrophil binding to endothelium via the upregulation of surface annexin‐A1.^45^ Of note, annexin‐A1 is also a critical mediator of glucocorticoid‐induced suppression of IL6 production and release in response to LPS via upregulation of the anti‐inflammatory factor GILZ (glucocorticoid‐induced leucine zipper), which directly represses NFκB and MAP kinase signaling.^46^ Intriguingly, given the high neutrophil burden present in draw 1 and the subsequent resolution of this burden by draw 2, dexamethasone‐induced annexin‐A1 expression may also have enhanced neutrophil apoptosis and increased monocytic efferocytosis of the dying granulocytes.^47^ While speculative, our experience with FIRES patients suggests that many exhibit abnormal neutrophil profiles that may reflect a failure in the resolution phase of the normal inflammatory response to infections within the middle ear or upper respiratory tract. The patient in this study presented with Streptococcal pharyngitis and it is possible that this infectious bacterial trigger led to the hyperinflammatory monocyte response and the large burden of neutrophils. Whether the failure of the patient's monocytes to respond to viral signals induces, contributes to, or amplifies the hyperinflammatory bacterial response remains to be determined.^48^ Future studies testing differential transcriptional and secretomic responses to bacterial and viral stimuli may reveal patient‐specific innate immunodeficiencies that predispose some children to FIRES in the context of prodromal bacterial infection, while other children are susceptible to FIRES as the result of a preceding viral infection. Such studies may also provide critical insights into the pathogenesis and pathophysiology of FIRES that may result in novel and individualized therapeutic approaches.

### Limitations

The current study was limited to only one FIRES patient. Future studies assessing the ex vivo response profile of multiple FIRES patients and identifying the potential impact of therapies such as intrathecal dexamethasone across subjects will be critically important. Another limitation to the current study was the absence of samples from the most hyper‐acute phase of disease and the inability to discern the potential effects of the first‐line therapies on the subsequent innate immune profile. Likewise, the inability to resolve the potential effect of high dose anakinra from the effect of intrathecal dexamethasone therapy limits the overall interpretation of our findings. Future efforts should strive to capture biofluid and cellular samples as soon as possible (ideally at presentation), although this approach is clearly complicated by the rapidly evolving identification of etiology in these patients that would likely result in a large amount of non‐FIRES sampling. Nonetheless, building a pipeline for immediate collection of biospecimens from all pediatric seizure cases that fail to respond to conventional anti‐seizure medications would provide unparalleled insight into the pathogenesis of FIRES and other refractory inflammatory/immune‐mediated seizure disorders.

## Conclusions

Despite limitations, several conclusions and interpretations can be drawn from our findings. First, the locus of corticosteroid delivery may matter more than the specific identity of the drug. Future studies should consider early intervention with continuous systemic dexamethasone or methylprednisolone infusion; alternatively, a single intrathecal dose of corticosteroid may be enough to reset the system and trigger resolution of an underlying hyperinflammatory state in FIRES patients. Employing such a therapy very early in the disease course in suspected FIRES cases may offer profound therapeutic efficacy with limited risk. Second, failure to resolve neutrophil responses to banal childhood infections may be a unifying mechanism in FIRES that should be addressed experimentally and therapeutically. Indeed, one explanation for the failure of single‐factor therapies such as tocilizumab or anakinra in some FIRES patients may be the absence of pro‐resolving drivers induced by such interventions. The multifaceted mechanistic effect of dexamethasone may be a key component in simultaneously reducing inflammatory drivers and restoring homeostasis in the monocyte‐neutrophil axis in FIRES. Third, measurement of inflammatory cytokines and chemokines in serum and CSF provides valuable insight into potential mechanisms and therapeutic targets in FIRES, but additional insight may be gleaned from including ex vivo response profiling. While logistically challenging, there may be considerable value in using such profiling to test specific therapeutic agents prior to initiating treatment in any given FIRES patient. Given the failure to date to identify a panacea for these patients, such an individualized approach may ultimately be necessary.

## Author Contributions

CLH and RFM conceived of the study. CLH designed the experiments. CLH, RFM, and NM interpreted the data. RKJ, BLO, and JAS performed experiments and acquired data. CLH wrote the initial draft of the manuscript. All authors edited and approved the submitted version.

## Conflict of Interest Statement

The authors declare that they have no competing interests.

## Data Availability

The datasets used and/or analyzed during the current study are available from the corresponding author upon reasonable request.
